# Earlier versus later initiation of renal replacement therapy among critically ill patients with acute kidney injury: a systematic review and meta-analysis of randomized controlled trials

**DOI:** 10.1186/s13613-017-0265-6

**Published:** 2017-04-05

**Authors:** Tai-Shuan Lai, Chih-Chung Shiao, Jian-Jhong Wang, Chun-Te Huang, Pei-Chen Wu, Eric Chueh, Shih-Chieh Jeff Chueh, Kianoush Kashani, Vin-Cent Wu

**Affiliations:** 1 Division of Nephrology, Department of Internal Medicine, National Taiwan University Hospital Bei-Hu Branch, No. 87, Neijiang St, Taipei, 108 Taiwan; 2 Community and Geriatric Research Center, National Taiwan University Hospital Bei-Hu Branch, No. 87, Neijiang St, Taipei, 108 Taiwan; 3 Division of Nephrology, Department of Internal Medicine, Saint Marys Hospital Luodong, No. 160, Zhongheng S. Rd., Luodong, Yilan, 26546 Taiwan, ROC; 4 Saint Mary’s Medicine, Nursing and Management College, No. 100, Ln. 265, Sec. 2, Sanxing Rd., Sanxing Township, Yilan County, 266 Taiwan, ROC; 5 Division of Nephrology, Department of Internal Medicine, Chi-Mei Medical Center, Liouying. No. 201, Taikang, Taikang Vil., Liuying Dist.736, Tainan City, Taiwan; 6 Division of Internal and Critical Care Medicine, Department of Critical Care Medicine, Taichung Veterans General Hospital, No. 1650 Taiwan Boulevard Sect. 4, Taichung, 40705 Taiwan; 7 Division of Nephrology, Department of Internal Medicine, Mackay Memorial Hospital, No. 92, Sec. 2, Zhongshan N. Rd., Taipei, 10449 Taiwan; 8 Case Western Reserve University, No. 10900 Euclid Ave., Cleveland, OH 44106 USA; 9 Cleveland Clinic Lerner College of Medicine and Glickman Urological and Kidney Institute, Cleveland Clinic, No. 9980, Carnegie Ave, Cleveland, OH 44195 USA; 10 Division of Nephrology and Hypertension, Department of Medicine, Mayo Clinic, No. 200 First St. SW, Rochester, MN 55905 USA; 11 Division of Pulmonary and Critical Care Medicine, Department of Medicine, Mayo Clinic, No. 200 First St. SW, Rochester, MN 55905 USA; 12 Division of Nephrology, Department of Internal Medicine, National Taiwan University Hospital, No. 7 Chung-Shan South Road, Zhong-Zheng District, Taipei, 100 Taiwan; 13 National Taiwan University Study Group on Acute Renal Failure (NSARF), Taipei, Taiwan

**Keywords:** Acute kidney injury, Length of stay, Meta-analysis, Mortality, Renal replacement therapy, Timing, CAKS, NSARF

## Abstract

**Background:**

Although the optimal timing of initiation of renal replacement therapy (RRT) in critically ill patients with acute kidney injury has been extensively studied in the past, it is still unclear.

**Methods:**

In this systematic review, we searched all related randomized controlled trials (RCTs) that directly compared earlier and later RRT published prior to June 25, 2016, from PubMed, MEDLINE, and EMBASE. We extracted the study characteristics and outcomes of all-cause mortality, RRT dependence, and intensive care unit (ICU) and hospital length of stay (LOS).

**Results:**

We identified 51 published relevant studies from 13,468 screened abstracts. Nine RCTs with 1627 participants were included in this meta-analysis. Earlier RRT was not associated with benefits in terms of mortality [relative risk (RR) 0.88, 95% confidence interval (CI) 0.68–1.14, *p* = 0.33] and RRT dependence (RR 0.81, 95% CI 0.46–1.42, *p* = 0.46). There were also no significant differences in the ICU and hospital LOS between patients who underwent earlier versus later RRT [standard means difference −0.08 (95% CI −0.26 to 0.09) and −0.11 (95% CI −0.37 to 0.16) day, respectively]. In subgroup analysis, earlier RRT was associated with a reduction in the in-hospital mortality among surgical patients (RR 0.78, 95% CI 0.64–0.96) and patients who underwent continuous renal replacement therapy (CRRT) (RR 0.80, 95% CI 0.67–0.96).

**Conclusions:**

Compared with later RRT, earlier initiation of RRT did not show beneficial impacts on patient outcomes. However, a lower rate of death was observed among surgical patients and in those who underwent CRRT. The included literature is highly heterogeneous and, therefore, potentially subject to bias. Further high-quality RCT studies are warranted.

**Electronic supplementary material:**

The online version of this article (doi:10.1186/s13613-017-0265-6) contains supplementary material, which is available to authorized users.

## Background

Acute kidney injury (AKI) is a common yet potentially fatal complication of illnesses among 1% of the community-based population, 8–15% of hospitalized patients, and up to 50% of critically ill patients admitted to the intensive care unit (ICU) [1–5]. AKI carries increased risk of morbidity and mortality and adds to the healthcare cost, even in mild temporary form [6–11].

Although renal replacement therapy (RRT) remains the primary supportive management strategy for patients with severe AKI, it could also be associated with complications and adverse events [12–14]. Despite improvements in RRT technology, it is still not clear whether the outcome of patients with AKI who require RRT has improved over the years [7, 15]. Earlier initiation of RRT may provide a better control of fluid and electrolyte balance, superior acid–base homeostasis, removal of uremic waste, and prevention of subsequent complications attributable to AKI [16]. Furthermore, earlier RRT could potentially limit the kidney-specific and remote organ injuries due to fluid overload, electrolyte imbalance, and systemic inflammation [17]. However, earlier RRT may also expose the patients to increased risks of hemodynamic instability, anticoagulation-induced bleeding, blood-stream infection, and even inflammatory or oxidative stress induced by the bio-incompatibility of the dialyzer membranes. In comparison, later initiation of RRT may allow more time for hemodynamic optimization prior to RRT, and it may avoid the need for RRT and its associated complications [18].

In recent decades, the timing of RRT initiation has been evaluated in different population types (e.g., surgical or medical patients). Variability in the definitions of AKI and RRT timing has resulted in contradicting conclusions among the various studies [19–23]. Similarly, previous systematic analyses regarding the optimal timing of RRT initiation were unable to draw definitive conclusions owing to the scarcity of large-scale randomized controlled trials (RCTs), non-standardized triggers for RRT initiation, and heterogeneities of population and study design. In summary, while the observational studies tended to show more beneficial effects for earlier RRT, clinical trials were unable to replicate these findings [24–27]. Recently, two large RCTs showed contradictory results and attracted considerable attention from both clinicians and researchers. The first was a multicenter RCT by the AKIKI study group [28], which showed no significant differences in 60-day mortality between early and delayed RRT groups. Another was the ELAIN trial, [29] a single-center RCT that showed significant benefits in terms of 90-day mortality, renal function recovery, and hospital length of stay (LOS) among patients in the early RRT group. Although these two RCTs exhibited opposing results, they added value to the field of critical care. This systematic review is conducted to include all relevant RCTs related to the impact of the timing of RRT initiation among critically ill patients with moderate to severe AKI.

## Methods

### Search strategy and selection criteria

In concordance with the Preferred Reporting Items for Systematic Reviews and Meta-analyses (PRISMA) guidelines, we conducted a systematic review and meta-analysis to investigate the effect of earlier initiation of RRT on the outcomes of critically ill patients with AKI who require dialysis [30]. We searched MEDLINE, PubMed, and EMBASE databases and identified the relevant articles published up to June 25, 2016, using Web of Science. We screened references by titles and abstracts and included related studies for further analysis. Case reports or case series, non-English articles, articles not focused on critically ill patients, studies consisting of pediatric patients, studies that did not present mortality data, and those that did not clearly define the timing of initiation of RRT were excluded. The keywords used for database search were provided in Additional file 1: Table S1. We only included studies with randomized controlled designs in the final meta-analysis. Both abstracts and full papers were selected for quality assessment and data syntheses. We contacted the authors of abstracts for further details, if available.

### Data extraction and synthesis

We extracted data regarding the year of publication and patient enrollment, leading author, the number of patients, and events from each article. When available, odds ratios and 95% confidence intervals (CIs) from these RCTs were extracted. Other parameters for record included the type of patient setting (surgical/mixed/medical), criteria used for AKI diagnosis, cohort size, presence of sepsis, study quality, and the proportions of patients on mechanical ventilation. Two researchers (TSL and CCH) independently extracted the data, and a third investigator (VCW) resolved any disagreements between them.

### Risk of bias assessment

We assessed the risk of bias in the included articles using structured assessment tools. For RCTs, we use Cochrane review tools to access the risk of bias [31]. We evaluated the adequacy of randomization and concealment, blinding, reporting of outcomes, sample size calculation, and disclosure of funding sources. We assessed the overall study quality according to current standards [31, 32].

### Definition of earlier versus later RRT initiation

The definition of earlier initiation of RRT varied substantially among included RCTs. In four of the included trials, initiation of RRT immediately after randomization was defined as “early” [28, 33–35]. In two studies, early initiation was considered when RRT started in less than 12 h of admission to ICU, while in another study, authors used the serum BUN > 70 mg/dL or creatinine >7 mg/dL and the defining criteria for RRT initiation [36, 37]. One study compared prophylactic hemodialysis before surgery with standard care [38]. In ELLAIN trial, early RRT was defined as initiation within 8 h of diagnosis of stage 2 AKI using the KDIGO classification [29]. We included all definition of early dialysis based on each individual study design in order to evaluate the potential effect of early dialysis on the primary outcome; obviously, this leads to increased heterogeneity observed in our analysis.

### Ascertainment of outcomes

The primary outcome of interest was all-cause mortality, including in-hospital mortality and 30-, 60-, and 90-day mortality. We also evaluated RRT dependence after hospital discharge. The secondary outcomes were ICU or hospital LOS.

### Statistical analyses

Owing to the significant heterogeneity among the enrolled studies, we used the random effects model. The overall summary risk ratios (RRs) and 95% CIs were calculated using the Mantel–Haenszel method. We characterized the heterogeneity with the *I* ^2^ and *τ* ^2^ statistic. A *p* value ≤0.05 was considered statistically significant. Sensitivity analyses were conducted for variables that could modify the effect of initiation time and mortality. In subgroup analysis, we performed meta-regression to assess the effect of interaction between variables and the timing of RRT initiation on mortality and RRT dependence. Funnel plots were drawn to evaluate the distribution of studies. Begg’s rank correlation test and Egger’s linear regression were used to assess the publication bias. We used STATA (version 13, Stata Corp. 2013. College Station, TX: Stata Corp LP), and Review Manager (RevMan) (version 5.2. Copenhagen: The Nordic Cochrane Centre, The Cochrane Collaboration, 2014) software for the meta-analysis.

## Results

### Study characteristics

**Fig. 1:**
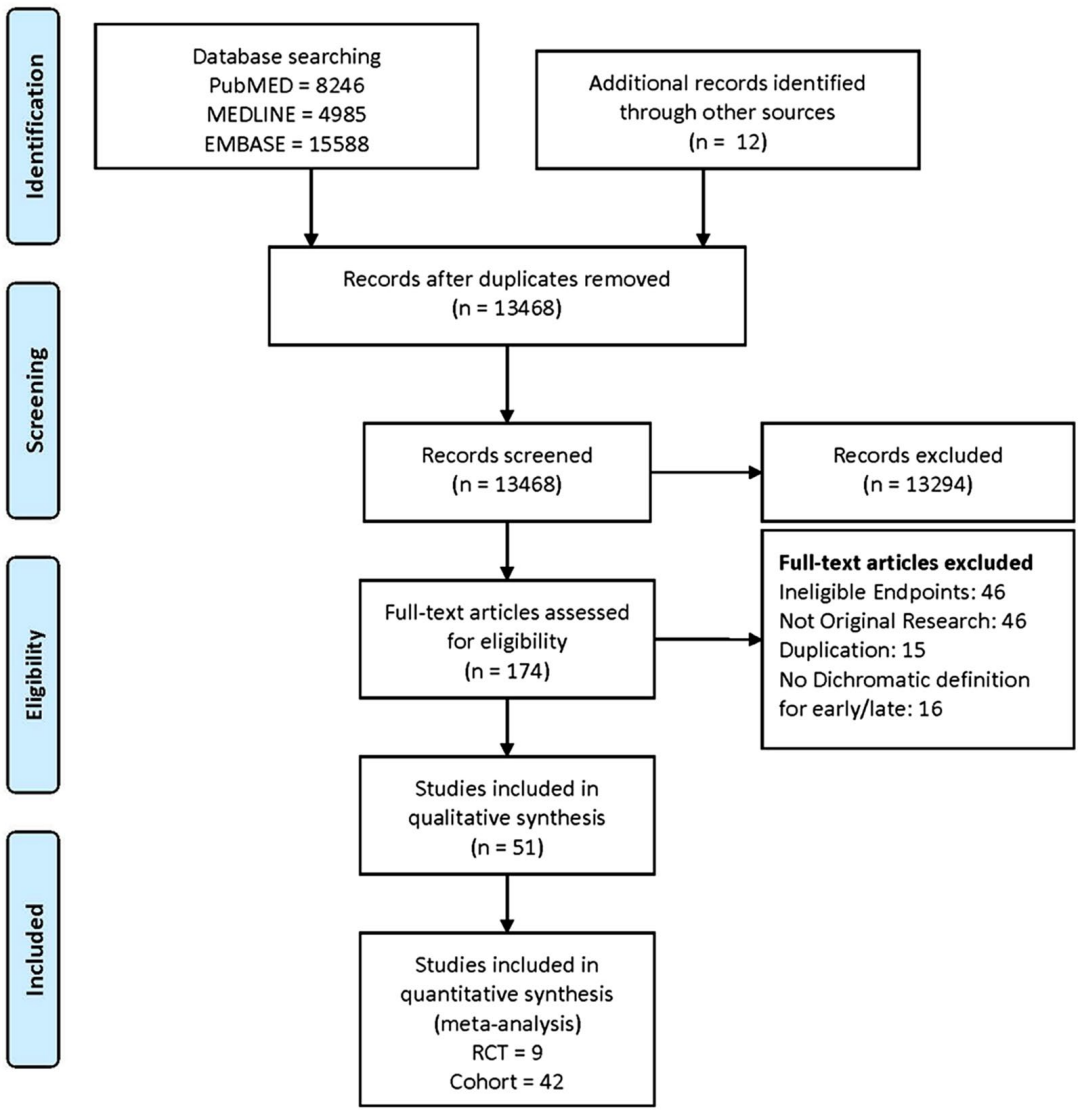
Flowchart of study selection for meta-analysis. *RCT* randomized controlled trials

Figure 1 shows the flowchart of the literature search and selection process. We screened 13,468 abstracts, of which 174 were eligible for full-text reviews. A total of 51 studies including 9 RCTs and 42 cohort studies presented data on the timing of RRT initiation among critically ill patients with AKI. These nine RCTs were included in the meta-analysis (Table 1). The trials were conducted in Europe, Asia, and Canada between 2002 and 2016.

**Table 1:** Summary of included randomized controlled trials

	References	Population setting	Study period	Nation	Sites^1^	Inclusion criteria	Exclusion criteria	n	Male	Mean Age	Mode	Sepsis	Endpoint	Counts
1	Bouman [53]	Mixed	Not specified	Netherland	M	Oliguria <30 cc/h, shock with vasopressor support	eGFR <30; cirrhosis obstructive AKI	71	42 (59%)	68.4	CRRT	n/a	In-hospital mortality	I: 18/35
														C: 14/36
													In-ICU mortality	I: 13/35
														C: 10/36
													28-day mortality	I: 11/35
														C: 9/36
2	Durmaz [38]	Surgical	n/a	Turkey	S	n/a	n/a	44	n/a	n/a	IHD	2 (4.5%)	In-hospital mortality	I: 1/20
														C: 7/16
3	Sugahara [33]	Surgical	1995–1997	Japan	S	Urine output < 30 cc/h or increase in SCr > 0.5 mg/dL/day	Pregnant Severe hepatic dysfunction (serum bilirubin level of 5.0 mg/dL or higher) Mental disorders Cancers	28	18 (64%)	64.5	CRRT	n/a	14-day mortality	I: 2/14
														C: 12/14
4	Koo [34]	Mixed	Not specified	Korea	S	Severe sepsis or septic shock	Not specified	102	62 (61%)	62	CRRT	102 (100%)	In-hospital mortality	I: 12/43
														C: 30/59
5	Jamale [37]	Mixed	2011–2012	India	S	Severe AKI with increasing BUN and SCr levels	Needing urgent dialysis; Previous dialysis history Recovering AKI	208	141 (68%)	42.4	IHD	44 (21%)	In-hospital mortality	I: 21//102
														C: 13/106
6	Combes [35]	Surgical	2009–2012	France	M	Postoperative shock, needing high dose vasopressors or ECMO	<18 years old; pregnant; Enrolled into current or other trials previous RRT Weight > 120 kg SAPSII > 90	224	177 (79%)	59.4	CRRT	n/a	In-hospital mortality	I: 50/112
														C: 44/112
													In-ICU mortality	I: 50/112
														C: 44/112
													30-day mortality	I: 40/112
														C: 40/112
													60-day mortality	I: 48/112
														C: 42/112
													90-day mortality	I: 51/112
														C: 43/112
7	Wald [36]	Mixed	2013–2013	Canada	M	KDIGO AKI Stage 3, oliguria > 12 h or NGAL > 400 ng/ml	Withdrawal of life support Intoxication RRT within the previous 2 months; renal obstruction, rapidly progressive glomerulonephritis, or interstitial nephritis; eGFR < 30 ml/min per 1.73 m^2^ the passage of 48 h since doubling of baseline serum creatinine	100	72 (72%)	63.1	Mixed	56 (56%)	In-hospital mortality	I: 16/48
														C: 19/52
													In-ICU mortality	I: 13/48
														C: 16/52
													90-day mortality	I: 18/48
														C: 19/52
8	Zarbock [29]	Surgical	2013–2015	Germany	S	KDIGO AKI stage 2	eGFR < 30 ml/min/1.732 m^2^ previous RRT AKI caused by permanent occlusion of renal artery or surgery Glomerular nephritis, interstitial nephritis, hemolytic uremic syndrome Pregnancy AIDS Heptorenal syndrome	231	146 (63%)	67.0	CRRT	75 (32%)	30-day mortality	I: 34/112
														C: 48/119
													60-day mortality	I: 43/112
														C: 60/119
													90-day mortality	I: 44/112
														C: 65/119
9	Gaudry [28]	Mixed	2013–2016	France	M	Ischemic or toxic AKI and receiving invasive mechanical ventilation, catecholamine infusion or both, and AKIN Stage 3	BUN > 112 mg/dL Blood *K* > 6.0 mmol/L A pH < 7.15 Acute pulmonary edema	619	405 (65%	66.2	Mixed	483 (78%)	30-day mortality	I: 129/311
														C: 134/308
													60-day mortality	I: 150/311
														C: 153/308

*I* Intervention group, *C* control group, *RCT* randomized controlled trial, *S* single-center study, *M* multicenter study, *n/a* not available, *BUN* blood urea nitrogen, *SCr* serum creatinine, *ECMO* extracorporeal membrane oxygenator, *AKI* acute kidney injury

A total of 1627 critically ill patients who underwent acute dialysis were enrolled in the final analysis. Seven of the nine studies provided quantifiable results for RRT dependence during the follow-up period. Four trials recruited surgical patients only, and two of them enrolled patients undergoing coronary bypass surgery. The remaining five trials enrolled patients in the mixed surgical/medical ICU setting. Continuous renal replacement therapy (CRRT) was used as the modality of choice in five trials, and two trials used intermittent hemodialysis (IHD). The remaining studies utilized a mixture of the two dialysis modalities. Early high-volume hemofiltration was compared with the standard of care in one RCT. The quality of the included RCTs varied; most of the studies lacked sufficient information regarding participants or personnel blinding and concealment processes (Additional file 1: Fig. S1). We divided these studies into high or low quality, with 3 out of 6 domains of bias as the cutoff for the quality assessment tool. Publication bias was tested, and funnel plot was drawn. There was no obvious impact of study sample size with a *p* = 0.846 for Egger’s test (Additional file 1: Fig. S2).

### Primary outcomes

**Fig. 2:**
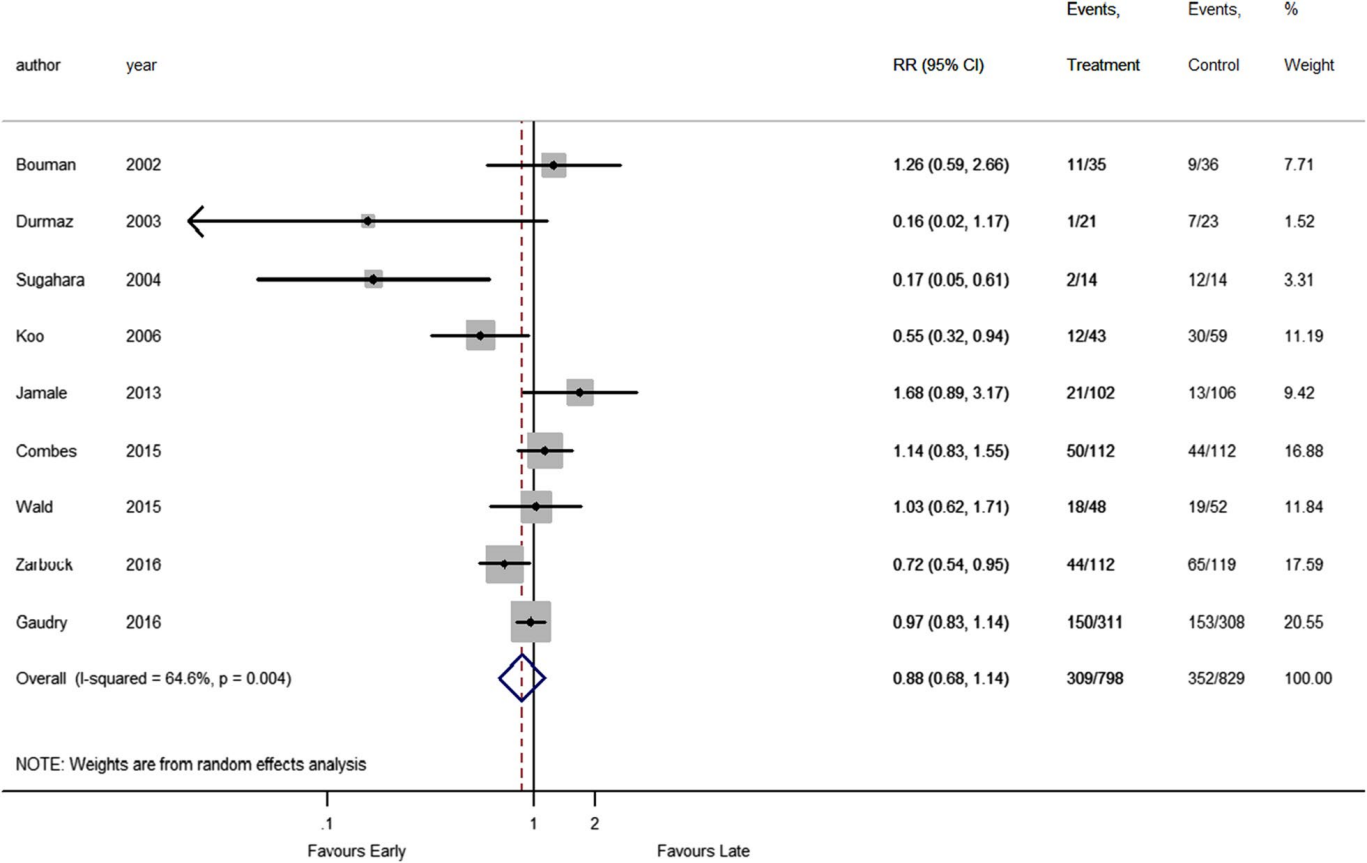
Forest plot for all-cause mortality: all studies. *RCT* randomized controlled trials, *RRT* renal replacement therapy

**Fig. 3:**
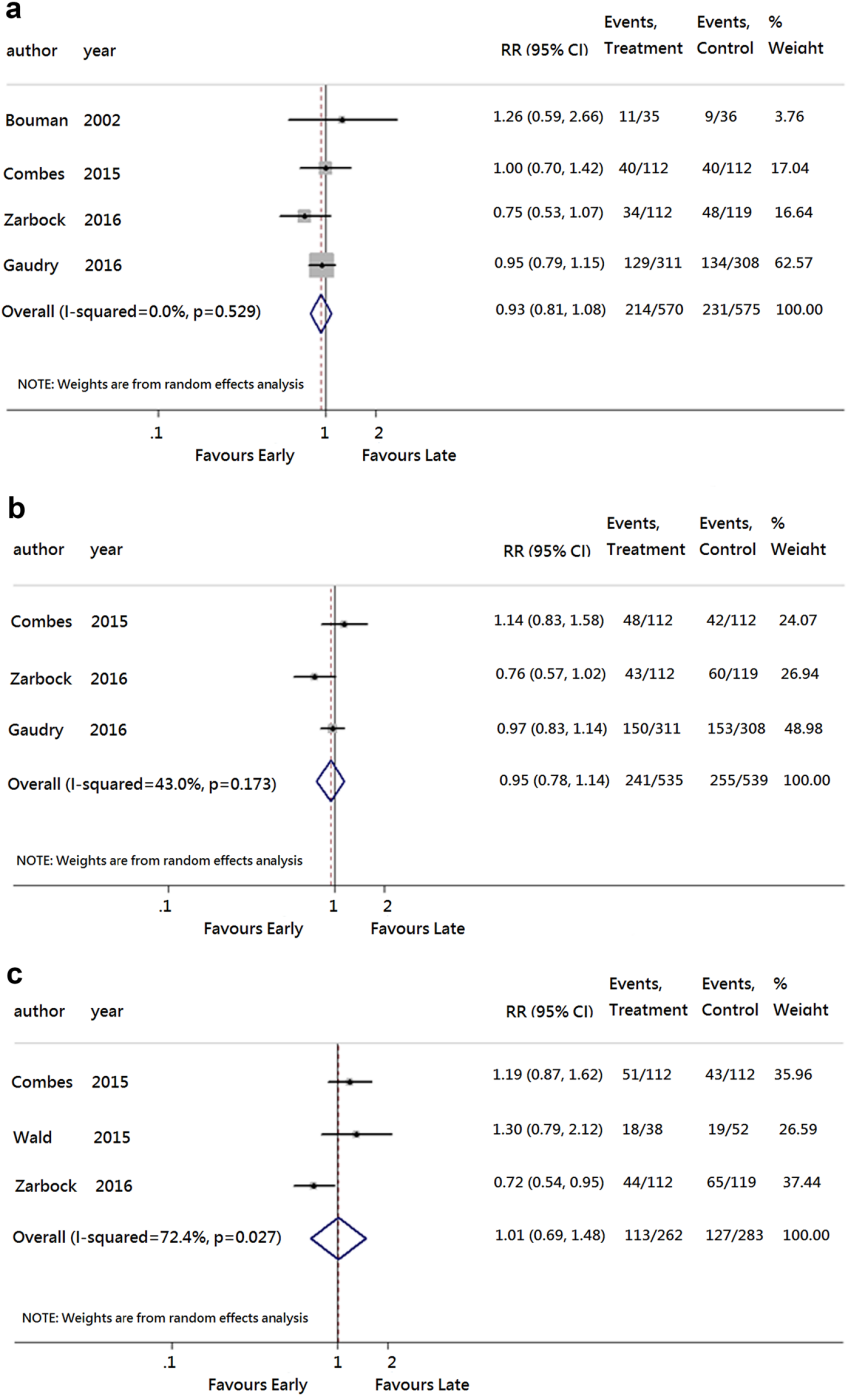
Forest plots for mortality in **a** 30 days, **b** 60 days, and **c** 90 days

Among the included trials, the pooled mortality rates were 38.7% (309 of 798) and 42.5% (352 of 829) in the groups of patients who underwent earlier and later RRT, respectively. Pooled estimates of included studies indicated no significant survival benefit in patients who underwent earlier RRT compared with those who underwent later RRT, with an RR of 0.88 (95% CI 0.68–1.14, *p* = 0.33) (Fig. 2). Substantial heterogeneity existed among studies, with an *I* ^2^ value of 64.6%, and Chi-square *p* = 0.004. In addition, there were no significant differences in the 30-, 60-, and 90-day mortality between groups with earlier and later RRT (Fig. 3).

**Fig. 4:**
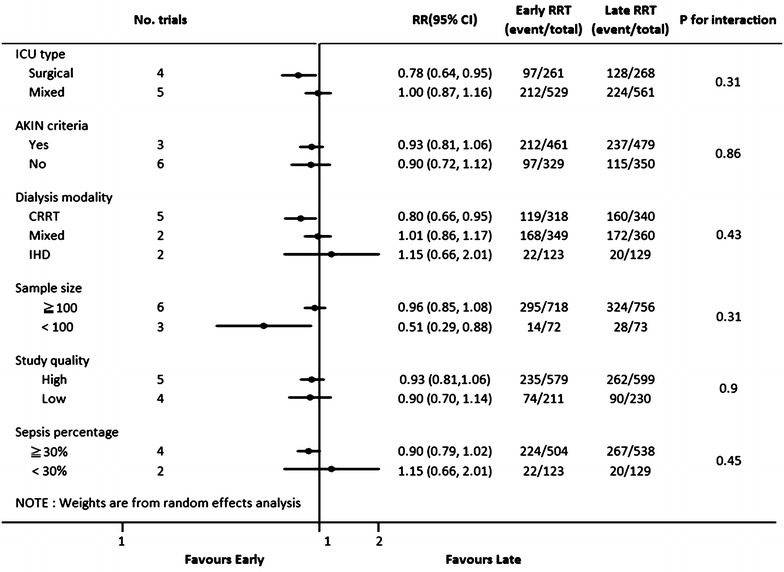
Forest plot for all-cause mortality: in subgroups. *RCT* randomized controlled trials, *RRT* renal replacement therapy

In the subgroup analyses, there were no differences between patients who underwent earlier and later RRT in the majority of subgroups, with three exceptions. Notably, earlier RRT seemed to provide a survival benefit in surgical patients (RR 0.78, 95% CI 0.64–0.95), but not in patients in the mixed ICUs (RR 1.00, 95% CI 0.87–1.16). This is despite the fact that we found no substantial evidence of such differences when trials were stratified by the ICU setting (*p* = 0.31 for interaction). Besides, the survival benefit of earlier RRT initiation was also observed in the patients who started with CRRT (RR 0.80, 95% CI 0.66–0.95), but not in those who received mixed modalities (RR 1.01, 95% CI 0.86–1.17) or IHD (RR 1.15, 95% CI 0.66–2.01) (*p* = 0.43 for interaction) (Fig. 4; Additional file 1: Fig. S3).

**Fig. 5:**
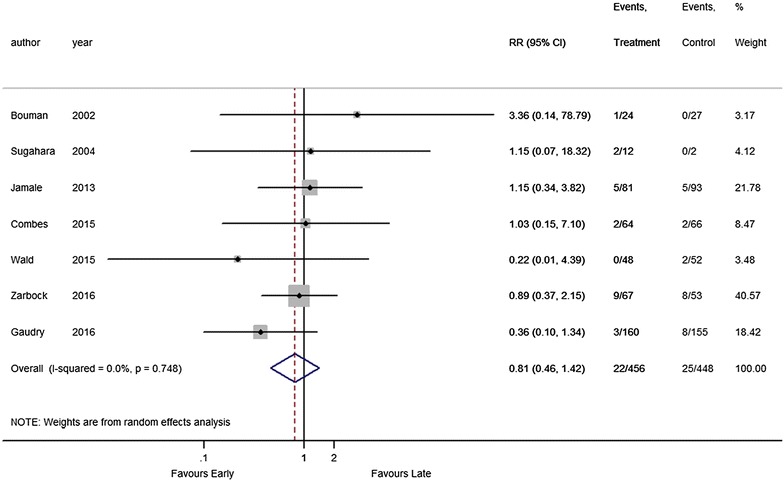
Forest plot for RRT dependence: all studies. *RCT* randomized controlled trials, *RRT* renal replacement therapy

Seven of the nine included RCTs reported information about RRT dependence. There was no statistically significant difference in the risk of RRT dependence between patients with earlier and later initiation of RRT, with a pooled RR of 0.81 (95% CI 0.46–1.42) (Fig. 5). There was no evidence for heterogeneity with an *I* ^2^ value = 0% and Chi-square *p* = 0.748). In the subgroup analysis, there was no statistically significant difference in RRT dependence across different subgroups (Additional file 1: Fig. S4).

### Secondary outcomes

The mean weighted ICU LOS was 12.5 days (*n* = 604) in the earlier RRT group and 13.0 days (*n* = 614) in the later RRT group. The mean weighted hospital LOS was 29.4 days (*n* = 604) in the earlier RRT group and 31.3 days (*n* = 614) in the later RRT group. Pooled analysis demonstrated no significant differences in the ICU LOS and hospital LOS between the two groups, with a standard difference in the means of −0.08 day (95% CI −0.26 to 0.09) and −0.11 day (95% CI −0.37 to 0.16), respectively (Additional file 1: Figs. S5, S6).

## Discussion

In the current systematic review of nine RCT including 1627 critically ill patients with AKI, who received RRT, we did not find any significant survival benefits in patients who underwent earlier versus later RRT. A considerable heterogeneity across studies was observed. Furthermore, 30-, 60-, and 90-day mortality, dialysis dependence, and LOS in the hospital or ICU were not lower in patients who underwent earlier RRT in comparison with those who underwent later RRT. We noticed a lower mortality rate in the earlier RRT group only in postsurgical patients and among those who underwent CRRT.

In recent interventional studies, no survival benefits have been observed among different intensities [39] and modalities of dialysis [40]. The optimal timing of RRT initiation still remains debatable owing to the contradictory reports in the literature. To our knowledge, the current systematic review is the first to exclusively include most RCTs to address the issue of the timing of RRT initiation and evaluate its impact on patient survival and RRT dependence (Table 2) [24–27, 41, 42].

**Table 2:** Comparisons of meta-analyses evaluating timing of RRT initiation in AKI and patients outcomes

	Population setting	Enrolled studies	Outcomes	Results (benefit of early RRT)	Limitations
Current study	Mixed patients with AKI (*n* = 1627)	Total nine RCTs. (publication date: 2002–2016)	In-hospital mortality; RRT dependence, 30-, 60-, 90-day mortality after hospital discharge	*All*: no significant advantage in survival (in-hospital, 30-, 60-, 90-day mortality) and RRT dependence *Subgroups*: Significant survival benefit in surgical patients and those started with CRRT	High heterogeneity among studies, varied definitions of early RRT
Seabra [41]	Mixed patients with AKI (*n* = 2378)	Total 23 studies including 4 RCTs, 1 quasi-RCTs, 1 prospective study, 16 retrospective studies, and 1 single-arm study (publication date: 1961–2006)	Mortality	*RCTs* nonsignificant 36% reduction of mortality risk *Cohort studies* significant 28% reduction of mortality risk (with significant heterogeneity among cohort studies) *Subgroups* smaller studies (*n* < 100) were more likely to show the benefit of early RRT	Paucity of RCTs, varied definitions of early RRT, many small sized studies, publications bias
Karvellas [26]	Mixed patients with AKI (*n* = 2684)	Total 15 studies including 2 RCTs, 4 prospective studies, and 9 retrospective studies (publication period: 1999–2010)	28-day mortality	*All* significant 55% reduction of mortality risk (with significant heterogeneity) *Subgroups* survival benefit remains in subgroup analysis according to ICU type, study design, and illness severity	Varied quality and high heterogeneity among studies Some studies were of small sample size Diverse definitions of early vs late RRT
Wang [42]	Mixed patients with AKI (*n* = 2955) About 50% studies involved surgical patients	Total 15 studies including 3 RCTs, 2 prospective studies, and 10 retrospective studies (publication period: 1990–2011)	Mortality	*All* significant 29% reduction of mortality risk (with high heterogeneity) *Subgroups* significant reduction of mortality risk of 31% in CRRT and 74% in IHD (without evidence of heterogeneity)	Many studies were of relative low quality, small sample size, diverse definitions of early vs late RRT
Liu [45]	Surgical patients with AKI (after cardiac surgery) (*n* = 841)	Total 11 studies including 2 RCTs and 9 retrospective studies (publication period: 1972–2011)	28-day mortality; ICU LOS	Significant 71% reduction of 28-day mortality risk and 3.9 days shorter ICU LOS (with high heterogeneity among studies)	Based on studies with various quality with very high heterogeneity of results
Wierstra [24]	Mixed patients with AKI (*n* = 1042)	Total nine high-quality studies including 6 RCTs, 1 prospective study, and 2 retrospective studies (publication period: 2002–2015)	1-month mortality; ICU/hospital LOS	*All* no significant advantage in survival and ICU/hospital LOS *Subgroups* no advantage in survival and ICU/hospital LOS	Statistically significant heterogeneity among studies Diverse definitions of early vs late RRT
Xu [25]	Mixed patients with AKI (*n* = 1257)	Total six RCTs (publication period: 2002–2016)	Mortality, renal recovery, composite endpoint	No difference in mortality, renal recovery, composite endpoint	Insufficient number of studies included, some RCTs were relatively small, diverse definitions of early vs late RRT

*AKI* acute kidney injury, *CRRT* continuous renal replacement therapy, *ICU* intensive care unit; *IHD* intermittent hemodialysis, *LOS* length of stay, *RCT* randomized controlled trial

### Subgroup analyses

We hypothesized that using consensus AKI definitions, enrolling sepsis-associated AKI, differences in sample sizes and study qualities had high impacts on patient outcomes observed among different investigations. When we used different AKI definitions, septic AKI, and study quality for subgroup analyses, we found no difference between earlier versus later RRT initiation time.

We found survival benefit for earlier RRT initiation when CRRT was utilized. Previous studies including one meta-analysis showed no difference in mortality or RRT dependence between various dialysis modalities [40, 43], while other meta-analyses showed that the use of CRRT decreases mortality or RRT dependence [42]. However, these findings largely were dependent on data from observational trials, which were potentially biased by allocation and the qualities were uncertain. Our analysis focused on RCTs, mostly with high qualities and appropriate randomization, and the results were more reliable. The possible mechanisms of the observed benefits from CRRT as the dialysis modality include gentler osmolar shifts, lower overall cumulative fluid balance, and clearance of inflammatory factors [44]. Our study is not able to identify the reasons behind improved outcomes with CRRT, and further studies are warranted.

We also reported a survival benefit for postsurgical critically ill patients with AKI when they received “earlier” RRT. A meta-analysis showed that early initiation of RRT for patients with AKI after cardiac surgery improved mortality [45]. Postoperative fluid overload in the surgical ICU is very common [46], and these patients may benefit from the earlier removal of excessive fluid by RRT [47, 48]. Other supporting evidence came from the observation that compared to patients admitted to medical ICU, those admitted to the surgical ICU admissions at a greater risk for aggravation of cardiovascular, neurological, and respiratory diseases [49]. The literature review indicated that following initial resuscitation in the postsurgical critical care setting, maintaining appropriate fluid balance through earlier RRT is clinically relevant [50]. Unlike surgical patients who often suffer from single organ failure, the heterogeneity of medical ICU patients may limit the effect of a single intervention (in this case “earlier” RRT). Additionally, many surgical patients who undergo elective surgeries have undergone extensive preoperative evaluation and optimization which contributed to their better outcomes in comparison with those of medical ICU patients [22].

In septic patients, earlier RRT was not found to be associated with improvement in mortality or RRT dependence. In this subgroup of patients, sepsis-associated AKI due to intrinsic renal lesions is only one part of the puzzle. Often, mortality in these patients correlates with various sepsis-induced inflammatory tissue damages and multi-organ failure [51]. Therefore, a single intervention may not be able to show a significant change in ICU outcomes, such as mortality. Furthermore, the possible adverse effects of earlier RRT such as enhanced clearance of antibiotics, amino acids and nutrients and hypothermia may counteract the benefits of a timely RRT. Moreover, in some studies, earlier initiation of RRT showed deleterious effects on the outcomes of patients with severe sepsis and septic shock; in addition, no differences were detected in their plasma cytokine levels [52]. Our results confirmed that earlier RRT initiation had no beneficial effects on the clinical outcomes of patients with sepsis-related AKI.

There have been previous systematic reviews consisting of a mixture of non-randomized cohort studies and a limited number of RCT regarding the optimal timing of RRT initiation [26, 27, 41, 42]. We were not able to confirm these reports in our systematic review. Previous studies concluded earlier RRT was associated with decreased mortality and RRT dependence in critically ill patients with AKI [26]. Contrary to the previous reports, we did not find a significant effect of earlier RRT on either ICU or hospital mortality and LOS, and dialysis dependence. In our study, only patients who underwent CRRT or postsurgical patients showed benefits in terms of the mortality rate for earlier RRT initiation.

One of the differences between the current study and previous reports was the inclusion of RCTs only, including the two latest published RCT studies [28, 29], which accounted for the different results of our study from those of the previously studies [24, 26, 27, 41]. Prior meta-analyses that concluded survival benefit attributed to earlier RRT initiation relied heavily on data from retrospective cohort studies that may possess incomplete pre-intervention data, preexisting significant differences among groups and heterogeneous study designs. Furthermore, observational studies are more subject to the selection bias when compared with RCTs.

As we showed, there is a significant heterogeneity among the studies related to the timing of RRT initiation which may impact the results we found in this systematic analysis. There are some possible explanations for the discordance and heterogeneity among different studies. Using varied definitions of AKI and different AKI stage criteria for RRT initiation accounted for part of the observed heterogeneity. In the majority of previously reported cohort studies, the differences in pre-intervention study groups contributed to the heterogeneity of the results, making the systematic reviews difficult to interpret. Furthermore, “patients without RRT” were not used as “control” in cohort studies. As illustrated by the AKIKI trial [28], the mortality in patients in the “delayed RRT” arm who never underwent RRT was lower than the mortality of patients who actually underwent RRT. Excluding patients who did not undergo RRT resulted in a significant bias. Another explanation is that compared with RCTs, observational studies (especially retrospective studies) are more subject to the selection bias. This highlights the critical need for a consensus definition of earlier versus later dialysis for the future studies and highlights the knowledge gap in the field. The STARRT-AKI study, a 2015 pilot trial that evaluated the feasibility and safety of early versus standard timing for starting RRT, will provide more evidence about the optimal timing of RRT initiation in AKI.

There are some limitations associated with our study. In our systematic review, we found no further information regarding the other factors associated with mortality; therefore, we cannot comment on the differences in the outcomes on the basis of a single intervention, i.e., earlier or later RRT initiation. Furthermore, no trial standardized the dialysis modality or dose delivered during RRT. We were not able to access the unpublished reports, which might have biased our results. Although our funnel meta-regression analysis showed a limited publication bias (Additional file 1: Fig. S2), the bias was always difficult to ascertain with a small sample number of the included studies. Finally, the definition of “earlier” RRT was variable and may have unduly influenced pooled effect estimates. As defined by traditional markers, RRT was initiated relatively late which may influence the effectiveness of the early treatment. Furthermore, in the majority of the enrolled studies, clinical patient care is individualized based on the discretion of the clinician. This would add to the heterogeneity of the studies and their results.

The strength of our present analysis rested on our extensive literature search on RCTs. We used standard Cochrane protocols and had the largest cumulative RCT study sample size in comparison with the previous reports. We only focused on the RCTs that had a reasonable quality with limited differential dropout based on the assigned treatment arm.

## Conclusion

Compared to later initiation of RRT, earlier RRT initiation in critically ill patients with AKI does not decrease mortality and long-term RRT dependence and does not alter the length of hospital stay. Earlier initiation of CRRT and earlier RRT in postsurgical patients may be associated with improved mortality. Future large-scale, multicenter, prospective interventional trials are needed to delineate the characteristics of patients who benefit from earlier initiation of RRT.

## Additional file

**Additional file 1.** Supplementary information.

## Abbreviations

AKI: acute kidney injury
CI: confidence interval
CRRT: continuous renal replacement therapy
ICU: intensive care unit
IHD: intermittent hemodialysis
LOS: length of stay
PRISMA: Preferred Reporting Items for Systematic Reviews and Meta-analyses
RCT: randomized controlled trial
RRT: renal replacement therapy
RR: risk ratio

## References

[1] Abelha FJ, Botelho M, Fernandes V, Barros H (2009). Determinants of postoperative acute kidney injury. Crit Care.

[2] Nisanevich V, Felsenstein I, Almogy G, Weissman C, Einav S, Matot I (2005). Effect of intraoperative fluid management on outcome after intraabdominal surgery. Anesthesiology.

[3] Clark E, Wald R, Walsh M, Bagshaw SM (2012). Timing of initiation of renal replacement therapy for acute kidney injury: a survey of nephrologists and intensivists in Canada. Nephrol Dial Transpl.

[4] Balk RA (2000). Severe sepsis and septic shock. Definitions, epidemiology, and clinical manifestations. Crit Care Clin.

[5] Stein A, de Souza LV, Belettini CR, Menegazzo WR, Viégas Jú R, Costa Pereira EM (2012). Fluid overload and changes in serum creatinine after cardiac surgery: predictors of mortality and longer intensive care stay. A prospective cohort study. Crit Care..

[6] Bagshaw SM, Laupland KB, Doig CJ, Mortis G, Fick GH, Mucenski M (2005). Prognosis for long-term survival and renal recovery in critically ill patients with severe acute renal failure: a population-based study. Crit Care.

[7] Uchino S, Kellum JA, Bellomo R, Doig GS, Morimatsu H, Morgera S (2005). Acute renal failure in critically ill patients: a multinational, multicenter study. JAMA.

[8] Manns B, Doig CJ, Lee H, Dean S, Tonelli M, Johnson D (2003). Cost of acute renal failure requiring dialysis in the intensive care unit: clinical and resource implications of renal recovery. Crit Care Med.

[9] Pannu N, James M, Hemmelgarn B, Klarenbach S (2013). Association between AKI, recovery of renal function, and long-term outcomes after hospital discharge. Clin J Am Soc Nephrol.

[10] Wu VC, Shiao CC, Chang CH, Huang TM, Lai CF, Lin MC (2014). Long-term outcomes after dialysis-requiring acute kidney injury. Biomed Res Int.

[11] Wu VC, Huang TM, Lai CF, Shiao CC, Lin YF, Chu TS (2011). Acute-on-chronic kidney injury at hospital discharge is associated with long-term dialysis and mortality. Kidney Int.

[12] Rondon-Berrios H, Palevsky PM (2007). Treatment of acute kidney injury: an update on the management of renal replacement therapy. Curr Opin Nephrol Hypertens.

[13] Schneider AG, Bellomo R, Bagshaw SM, Glassford NJ, Lo S, Jun M (2013). Choice of renal replacement therapy modality and dialysis dependence after acute kidney injury: a systematic review and meta-analysis. Intensive Care Med.

[14] Akhoundi A, Singh B, Vela M, Chaudhary S, Monaghan M, Wilson GA (2015). Incidence of adverse events during continuous renal replacement therapy. Blood Purif.

[15] Mehta RL, Pascual MT, Soroko S, Savage BR, Himmelfarb J, Ikizler TA (2004). Spectrum of acute renal failure in the intensive care unit: the PICARD experience. Kidney Int.

[16] Wald R, Bagshaw SM (2014). The timing of renal replacement therapy initiation in acute kidney injury: is earlier truly better?. Crit Care Med.

[17] Gibney N, Hoste E, Burdmann EA, Bunchman T, Kher V, Viswanathan R (2008). Timing of initiation and discontinuation of renal replacement therapy in AKI: unanswered key questions. Clin J Am Soc Nephrol.

[18] Shingarev R, Wille K, Tolwani A (2011). Management of complications in renal replacement therapy. Semin Dial.

[19] Carl DE, Grossman C, Behnke M, Sessler CN, Gehr TWB (2010). Effect of timing of dialysis on mortality in critically ill, septic patients with acute renal failure. Hemodial Int..

[20] Wu VC, Ko WJ, Chang HW, Chen YS, Chen YW, Chen YM (2007). Early renal replacement therapy in patients with postoperative acute liver failure associated with acute renal failure: effect on postoperative outcomes. J Am Coll Surg.

[21] Elahi MM, Lim MY, Joseph RN, Dhannapuneni RRV, Spyt TJ (2004). Early hemofiltration improves survival in post-cardiotomy patients with acute renal failure. Eur J Cardiothorac Surg..

[22] Shiao CC, Ko WJ, Wu VC, Huang TM, Lai CF, Lin YF (2012). U-curve association between timing of renal replacement therapy initiation and in-hospital mortality in postoperative acute kidney injury. PLoS ONE.

[23] Shiao CC, Wu VC, Li WY, Lin YF, Hu FC, Young GH (2009). Late initiation of renal replacement therapy is associated with worse outcomes in acute kidney injury after major abdominal surgery. Crit Care.

[24] Wierstra BT, Kadri S, Alomar S, Burbano X, Barrisford GW, Kao RLC (2016). The impact of “early” versus “late” initiation of renal replacement therapy in critical care patients with acute kidney injury: a systematic review and evidence synthesis. Crit Care.

[25] Xu Y, Gao J, Zheng X, Zhong B, Na Y, Wei J (2016) Timing of initiation of renal replacement therapy for acute kidney injury: a systematic review and meta-analysis of randomized-controlled trials. Clin Exp Nephrol. [Epub ahead of print]. 10.1007/s10157-016-1316-2 27485542

[26] Karvellas CJ, Farhat MR, Sajjad I, Mogensen SS, Leung AA, Wald R (2011). A comparison of early versus late initiation of renal replacement therapy in critically ill patients with acute kidney injury: a systemic review and meta-analysis. Crit Care.

[27] Crews DC, Scialla JJ, Liu J, Guo H, Bandeen-Roche K, Ephraim PL (2014). Predialysis health, dialysis timing, and outcomes among older United States adults. J Am Soc Nephrol.

[28] Gaudry S, Hajage D, Schortgen F, Martin-Lefevre L, Pons B, Boulet E (2016). Initiation strategies for renal-replacement therapy in the intensive care unit. N Eng J Med..

[29] Zarbock A, Kellum JA, Schmidt C, Van Aken H, Wempe C, Pavenstädt H (2016). Effect of early vs delayed initiation of renal replacement therapy on mortality in critically ill patients with acute kidney injury: The ELAIN Randomized Clinical Trial. JAMA.

[30] Moher D, Liberati A, Tetzlaff J, Altman DG, Group P (2009). Reprint–preferred reporting items for systematic reviews and meta-analyses: the PRISMA statement. Phys Ther.

[31] Higgins JP, Thompson SG, Deeks JJ, Altman DG (2003). Measuring inconsistency in meta-analyses. BMJ.

[32] Juni P, Witschi A, Bloch R, Egger M (1999). The hazards of scoring the quality of clinical trials for meta-analysis. JAMA.

[33] Sugahara S, Suzuki H (2004). Early start on continuous hemodialysis therapy improves survival rate in patients with acute renal failure following coronary bypass surgery. Hemodial Int.

[34] Koo JR, Yoon JW, Oh JE, Lee YK, Kim SG, Seo JW (2006). Prospective evaluation of early continuous venovenous hemofiltration (CVVH) on the outcome in patients with severe sepsis or septic shock. J Am Soc Nephrol.

[35] Combes A, Brechot N, Amour J, Cozic N, Lebreton G, Guidon C (2015). Early high-volume hemofiltration versus standard care for post-cardiac surgery shock. The HEROICS Study. Am J Respir Crit Care Med.

[36] Wald R, Adhikari NK, Smith OM, Weir MA, Pope K, Cohen A (2015). Comparison of standard and accelerated initiation of renal replacement therapy in acute kidney injury. Kidney Int.

[37] Jamale TE, Hase NK, Kulkarni M, Pradeep KJ, Keskar V, Jawale S (2013). Earlier-start versus usual-start dialysis in patients with community-acquired acute kidney injury: a randomized controlled trial. Am J Kidney Dis.

[38] Durmaz I, Yagdi T, Calkavur T, Mahmudov R, Apaydin AZ, Posacioglu H (2003). Prophylactic dialysis in patients with renal dysfunction undergoing on-pump coronary artery bypass surgery. Annals Thorac Surg..

[39] Palevsky PM, Zhang JH, O’Connor TZ, Chertow GM, Crowley ST, Choudhury D, VA/NIH ARFT Network (2008). Intensity of renal support in critically ill patients with acute kidney injury. N Eng J Med..

[40] Bagshaw SM, Berthiaume LR, Delaney A, Bellomo R (2008). Continuous versus intermittent renal replacement therapy for critically ill patients with acute kidney injury: a meta-analysis. Crit Care Med.

[41] Seabra VF, Balk EM, Liangos O, Sosa MA, Cendoroglo M, Jaber BL (2008). Timing of renal replacement therapy initiation in acute renal failure: a meta-analysis. Am J Kidney Dis.

[42] Wang X, Jie Yuan W (2012). Timing of initiation of renal replacement therapy in acute kidney injury: a systematic review and meta-analysis. Ren Fail.

[43] Mehta RL, McDonald B, Gabbai FB, Pahl M, Pascual MT, Farkas A, Kaplan RM (2001). Collaborative Group for Treatment of ARFitICU. A randomized clinical trial of continuous versus intermittent dialysis for acute renal failure. Kidney Int.

[44] Kashani K, Mehta RL (2016). We restrict CRRT to only the most hemodynamically unstable patients. Semin Dial.

[45] Liu Y, Davari-Farid S, Arora P, Porhomayon J, Nader ND (2014). Early versus late initiation of renal replacement therapy in critically ill patients with acute kidney injury after cardiac surgery: a systematic review and meta-analysis. J Cardiothorac Vasc Anesth.

[46] Lowell JA, Schifferdecker C, Driscoll DF, Benotti PN, Bistrian BR (1990). Postoperative fluid overload: not a benign problem. Crit Care Med.

[47] Bagshaw SM, Uchino S, Bellomo R, Morimatsu H, Morgera S, Schetz M (2009). Timing of renal replacement therapy and clinical outcomes in critically ill patients with severe acute kidney injury. J Crit Care.

[48] Smak Gregoor PJ, van Saase JL, vd Ingh HF, Weimar W, Kramer P (1996). Disseminated histoplasmosis in a haemodialysis patient on immunosuppression after graft failure. Nephrol Dial Transpl.

[49] Singbartl K, Kellum JA (2012). AKI in the ICU: definition, epidemiology, risk stratification, and outcomes. Kidney Int.

[50] Xu J, Shen B, Fang Y, Liu Z, Zou J, Liu L (2015). Postoperative fluid overload is a useful predictor of the short-term outcome of renal replacement therapy for acute kidney injury after cardiac surgery. Medicine..

[51] Bagshaw SM, Uchino S, Bellomo R, Morimatsu H, Morgera S, Schetz M (2007). Septic acute kidney injury in critically ill patients: clinical characteristics and outcomes. Clin J Am Soc Nephrol.

[52] Payen D, Mateo J, Cavaillon JM, Fraisse F, Floriot C, Vicaut E (2009). Impact of continuous venovenous hemofiltration on organ failure during the early phase of severe sepsis: a randomized controlled trial. Crit Care Med.

[53] Bouman CSC, Oudemans-Van Straaten HM, Tijssen JGP, Zandstra DF, Kesecioglu J (2002). Effects of early high-volume continuous venovenous hemofiltration on survival and recovery of renal function in intensive care patients with acute renal failure: a prospective, randomized trial. Crit Care Med.

