# Supplementary material for: Earlier versus later initiation of renal replacement therapy among critically ill patients with acute kidney injury: a systematic review and meta-analysis of randomized controlled trials
Source: Ann Intensive Care. 2017 Apr 5;7:38. doi: 10.1186/s13613-017-0265-6 (PMC5382114; doi:10.1186/s13613-017-0265-6)
Supplement: Supplementary file 1 — Additional file 1. Supplementary information. [file 13613_2017_265_MOESM1_ESM.docx]

**Earlier versus later initiation of renal replacement therapy for acute kidney injury: a systematic review and meta-analysis of randomized controlled trials**

**FIGURES AND TABLES:**

**Figure S1. The risk of bias assessment of included RCTs.**

Abbreviations: RCT: randomized controlled trials.

**
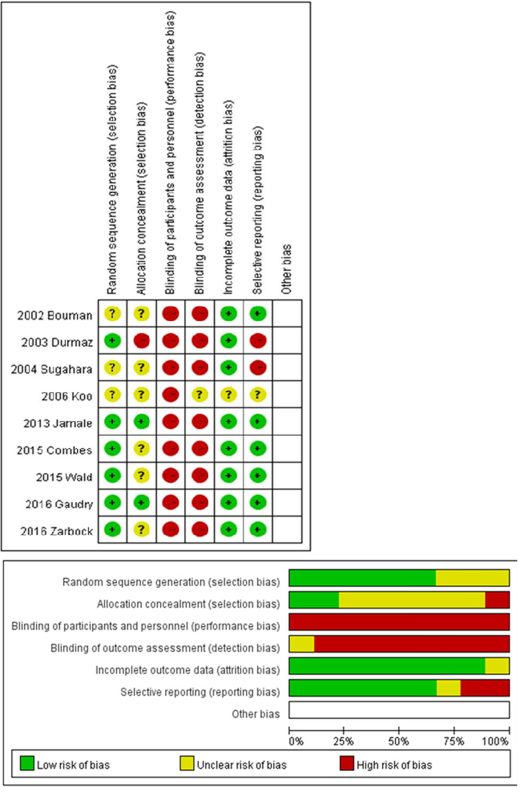
**

**Figure S2. Funnel plots for publication bias among included RCTs.**

Note: X-axis is RR of all-cause mortality. Y-axis is standard error of log RR.

Abbreviations: RCT, randomized controlled trial; RR, relative risk.


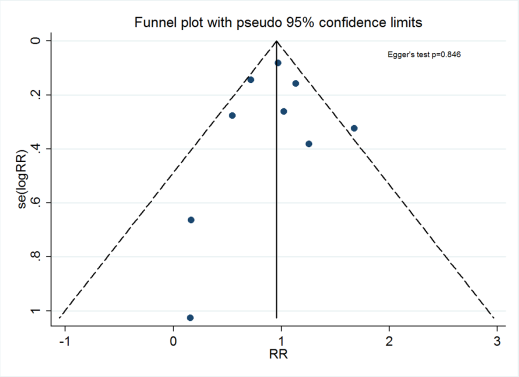


**Figure S3. Meta-regression plots for all-cause mortality by different settings.**

Note: (a) ICU settings and (b) dialysis modalities. The plots showed that the risk for all-cause mortality was lower in surgical ICU as comparing with mixed ICU, and CRRT as comparing with IHD and mixed dialysis modality.

Abbreviations: CRRT, continuous renal replacement therapy; ICU, intensive care unit; IHD, intermittent hemodialysis; RCT, randomized controlled trial.

**
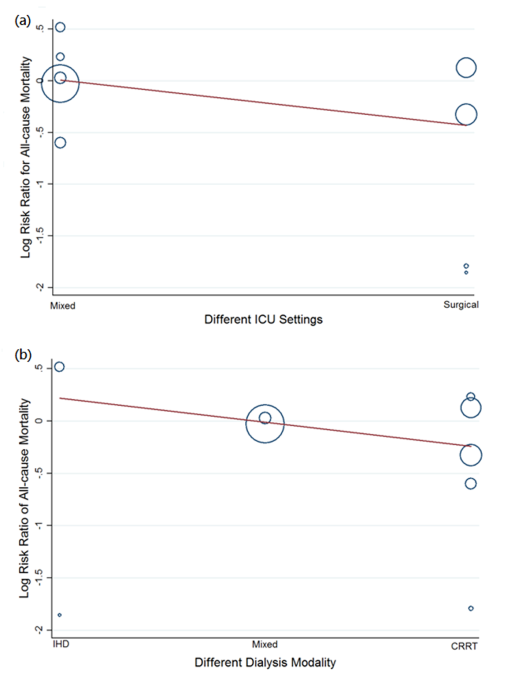
**

**Figure S4. Forest plot for RRT dependence: in subgroups.**

**Abbreviations: RRT, renal replacement therapy.**

**
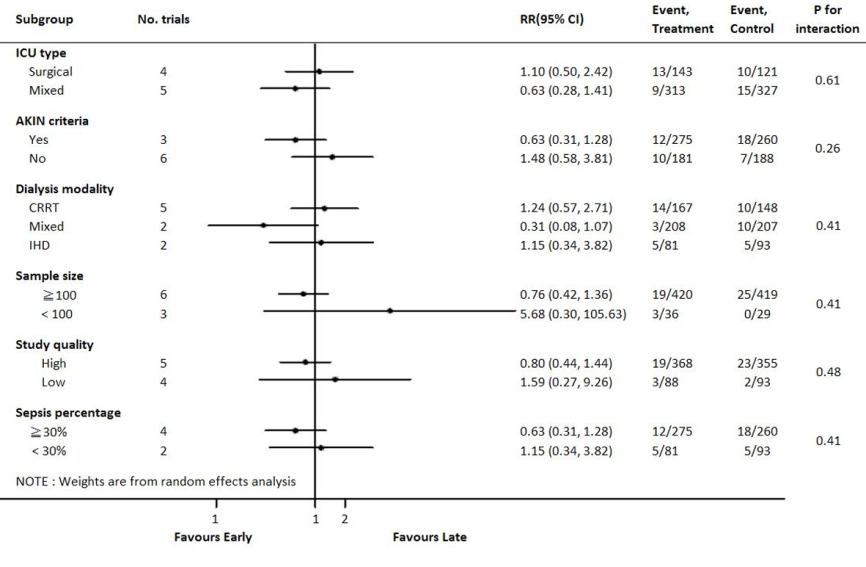
**

**Figure S5. Forest plot for ICU LOS in subgroups.**

Note: (a) stratified by ICU settings, and (b) stratified by dialysis modalities. Early RRT initiation was not associated with shorter length of stay in ICU compared to late initiation among patients in both mixed ICU (pooled SMD-0·02; 95% CI -0·17 to 0·13) and surgical ICU (pooled SMD -0·19; 95% CI -0·56 to 0·19), as well as those received CRRT (pooled SMD -0·00; 95% CI -0·19 to 0·18), and mixed modality (pooled SMD -0·02; 95% CI -0·17 to 0·13). However, early RRT initiation demonstrated benefit in shorter length of ICU stay in patients receiving IHD (only one study) (SMD -0·88; 95% CI -1·51 to -0·26).

Abbreviations: CI, confidence interval; CRRT, continuous renal replacement therapy; ICU, intensive care unit; IHD, intermittent hemodialysis; RCT, randomized controlled trial; RRT, renal replacement therapy ; SMD, standardized median difference.

**
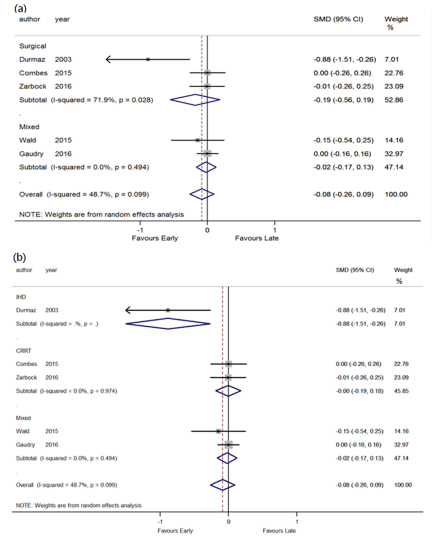
**

**Figure S6. Forest plot for hospital LOS in subgroups.**

Note: (a) stratified by ICU settings, and (b) stratified by dialysis modalities. Early RRT initiation was not associated with shorter length of stay in hospital compared to late initiation among patients in both mixed ICU (pooled SMD -0·12; 95% CI -0·27 to 0·03) and surgical ICU (pooled SMD -0·16; 95% CI -0·71 to 0·39), as well as those received CRRT (pooled SMD 0·05; 95% CI -0·55 to 0·65), and mixed modality (pooled SMD -0·12; 95% CI -0·27 to 0·03). However, early RRT initiation demonstrated benefit in shorter length of hospital stay in patients receiving IHD (only one study) (SMD -0·71; 95% CI -1·33 to -0·10).

Abbreviations: CI, confidence interval; CRRT, continuous renal replacement therapy; ICU, intensive care unit; IHD, intermittent hemodialysis; RCT, randomized controlled trial; RRT, renal replacement therapy; SMD, standardized median difference.


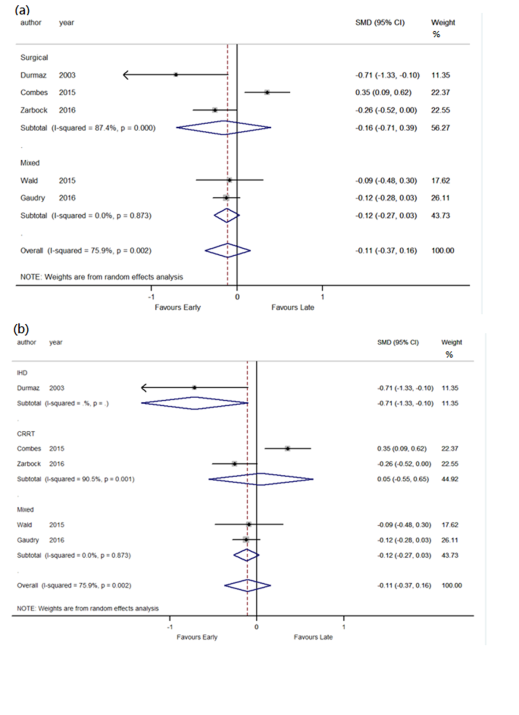


**Table S1. Keywords for database search.**

| #1. | ( Dialysis) OR (Renal Replacement Therapy) OR (Hemodialysis) OR (hemofiltration) OR (Hemodiafiltration) |
| --- | --- |
| #2. | (Acute kidney Injury) OR (acute renal failure) OR (Anuria) OR (Oliguria) OR (Acidosis) |
| #3. | (Timing) OR (Time) OR (Start) OR (Initiation) OR (Begin) OR (Early) OR (Late) OR (Beginning) OR (Commencement) |
| #4. | (#1) AND (#2) AND (#3) |
